# Factors Influencing Compliance With Personal Protective Equipment (PPE) Use Among Healthcare Workers

**DOI:** 10.7759/cureus.35269

**Published:** 2023-02-21

**Authors:** Jisa George, Naseema Shafqat, Ranjana Verma, Anurag Bhai Patidar

**Affiliations:** 1 Medical Surgical Nursing (Oncology Nursing), All India Institute of Medical Sciences, Bhopal, Bhopal, IND; 2 Obstetrics & Gynaecology, All India Institute of Medical Sciences, Bhopal, Bhopal, IND; 3 Nursing, All India Institute of Medical Sciences, Bhopal, Bhopal, IND; 4 Medical Surgical Nursing (Cardiothoracic and Vascular Nursing), All India Institute of Medical Sciences, Bhopal, Bhopal, IND

**Keywords:** safety equipment, ppe, health care workers, factors, personal protective equipment, compliance

## Abstract

Introduction

Accurate and appropriate use of personal protective equipment (PPE) is an integral component in infection prevention and control policy to ensure healthcare workers’ safety. Poor compliance with personal protective behaviours and inconsistent use of PPE has been identified as the main cause of transmission of nosocomial infections in healthcare settings and this reduced compliance is linked to many individual, environmental, and organizational factors. Therefore, the current study was carried out to identify various factors influencing PPE use among healthcare workers.

Materials and methods

A descriptive cross-sectional survey has been carried out among healthcare workers selected from two selected tertiary care hospitals in central India. Data on compliance with PPE and factors influencing compliance were collected using a three-point rating scale and structured questionnaire. Quantile regression was performed to identify the factors associated with adherence to PPE use among healthcare workers.

Results

The median score for compliance with PPE use among healthcare workers was found to be 22 with an interquartile range (IQR) of 16-24. The multiple quantile regression found that variables such as occupation (p<0.001), institutional policy (p=0.003), quality of PPE (p=0.002), availability of PPE (p<0.001), and improper size (p=0.042) were significantly associated with PPE compliance by healthcare workers.

Conclusion

The current study highlights the importance of taking adequate measures by the government and healthcare organizations to eliminate various factors hindering PPE compliance levels among healthcare workers to ensure consistent use of PPE by healthcare workers to safeguard themselves and patients.

## Introduction

Personal protective equipment (PPE) is designed to protect healthcare providers from serious workplace injuries, illnesses, and hospital-acquired infections. Appropriate use of PPE is considered an important strategy in infection prevention and control (IPC) policy and protects healthcare workers (HCWs) from acquiring infections with harmful pathogens. PPE was widely used to ensure the safety of HCWs during the outbreaks of various infectious diseases like severe acute respiratory syndrome (SARS), Middle East respiratory syndrome (MERS), Ebola virus, and swine flu. World Health Organization (WHO) guidelines recommended PPE use as a part of comprehensive infection prevention and control strategy during the recent pandemic outbreak of coronavirus disease 2019 (COVID-19) to ensure the safety of HCWs [1-3]. Poor compliance with personal protective behaviours, improper use of PPE, and reuse of PPE were associated with an increased risk of infection among frontline HCWs during the COVID-19 pandemic [4,5]. Besides this, reduced chances of getting infections were reported among HCWs using PPE appropriately as per the guidelines [6]. Prolonged exposure to infected persons without adopting adequate infection control practices and protective measures was associated with an increased risk of infection among HCWs [7]. A recent study carried out during the outbreak of COVID-19 reported a high incidence rate of infection due to underutilization of PPE [8].

Despite the emphasis given to various policies and guidelines on PPE use, inconsistent use of PPE has been reported among HCWs and it remains a greater challenge. Many studies have reported very low compliance with PPE use among HCWs and compliance with PPE use varies from individual to individual. Studies carried out to identify factors affecting adherence to IPC guidelines during respiratory outbreaks suggested that protective practices are influenced by a clear understanding of the guidelines, support received from managers and supervisors, communication about guidelines, sufficient resources, the perceived value of following guidance, the comfort of personal protective equipment (PPE), and availability of resources [5,6,9,10]. HCWs need adequate PPE knowledge and skill including that for appropriate selection, donning, removal, decontamination, and disposal of PPE for effective protection in clinical areas. Joint efforts by individual healthcare personnel, managers, and institutions are needed to improve the safety culture in healthcare facilities [11]. Commitment and support at all levels are essential to encourage and enforce appropriate PPE use and safety of HCWs. It is very important to identify barriers and facilitators influencing PPE use among HCWs and existing gaps in the implementation of various strategies and guidelines to develop interventions to improve compliance with PPE use. Identification of potential barriers and challenges and incorporating and assimilating this into the PPE policies and work culture of the organization eliminates barriers and improves critical thinking abilities to use PPE appropriately. Therefore, the current study was carried out to assess various factors influencing compliance with PPE use among HCWs and to provide baseline data for developing robust guidelines and protocols on the rational use of PPE to fight against future pandemic outbreaks.

## Materials and methods

A quantitative descriptive design was adopted in the current study to assess various factors affecting compliance with PPE use among HCWs. Study participants included 301 frontline HCWs working at the All India Institute of Medical Sciences (AIIMS), Bhopal, India, and Gandhi Medical College, Bhopal, India. The study was carried out from July 2021 to December 2021. HCWs with more than six months of experience in the selected healthcare institutions, who understood English or Hindi, and who were willing to participate were included in the study. Ethical clearance to carry out the study was obtained from the Institutional Human Ethics Committee of All India Institute of Medical Sciences, Bhopal, India (approval number: IHEC-LOP/2021/ IM0363). Written informed consent was taken from each participant before the collection of data. Participation in the study was voluntary, anonymity was maintained, and confidentiality of the collected information was assured.

The sample size for the study was estimated based on the findings of the pilot study. The minimum required sample size was calculated based on the sample size calculation formula for multiple linear regression with an anticipated correlation coefficient of 0.3 and multiple correlation coefficient value of 0.7 using power analysis with a statistical power of 90% and level of significance at 0.05 level. The minimum estimated sample size was found to be 265. Convenient sampling technique was used to recruit participants for the study.

Data was collected using a sociodemographic data sheet to assess the demographic variables of the study subjects, a three-point rating scale to assess the level of compliance with PPE use, a five-point Likert scale to assess the attitude of HCWs towards PPE use, and a structured questionnaire to assess factors influencing compliance with PPE use. Self-reported data on compliance with PPE use and factors influencing compliance were collected from the HCWs. All the data collection tools were developed by researchers based on the literature review and objectives of the study. The sociodemographic data sheet comprised eight items to assess the demographic characteristics of the participants. The rating scale to assess compliance with PPE use by HCWs included 12 items and each item was rated on a three-point rating scale of 'never', 'sometimes', and 'always' with ratings of 0, 1, and 2, respectively with a total score ranging from 0-24. The Likert scale to assess the attitude of the HCWs towards PPE use consisted of 10 items with a total minimum and maximum score of 10 and 50 respectively. The questionnaire to assess factors influencing compliance with PPE use included seven facilitating factors and 18 hindering factors with dichotomous options as ‘Yes’ or ‘No’. The content validity of the data collection tool was obtained from a panel of experts. The content validity index was calculated and was found to be appropriate. Cronbach alpha was calculated to check for internal consistency and the value was found to be 0.86 for the rating scale to assess the level of compliance with PPE use and 0.78 for the attitude scale to measure the attitude of HCWs regarding PPE use.

Data was collected through Google Forms (Google LLC, Mountain View, California, United States) either sent via email or WhatsApp (Whatsapp LLC, Menlo Park, California, United States). Participants who met inclusion and exclusion criteria were invited to participate in the survey and the invitation along with the link to the questionnaire was sent to the target participants. Collected data were analyzed using appropriate descriptive and inferential statistics with SPSS for Windows, Version 16.0 (Released 2007; SPSS Inc., Chicago, United States). The normality of the data was assessed using the Kolmogorov-Smirnov test (KS) test. All the categorical variables are summarised using frequency and percentage. Quantitative variables are summarized using median and interquartile range (IQR), Q1, and Q3, as data violates the normality assumptions. Quantile regression was performed to identify the factors associated with compliance with PPE use as the outcome variable violates the normality assumption. Variables that are found to be statistically significant (p< 0.05) in the univariate quantile regression were selected for the multiple quantile regression model.

## Results

Most of the subjects (n=222, 73.8%) were below 30 years of age and were males (n=206, 68.4%). The median age of the participants was found to be 28 years with an IQR of 27-31. While considering occupation, the highest proportion of participants (n=220, 73.1%) was nursing personnel. With respect to the area of work, the highest number of participants (n=143, 47.5%) was working in general wards. While considering work experience, a large proportion of participants (n=160, 53.2%) had an experience between one to five years with a median of three years (IQR of 2-6). A description of the demographic characteristics of the study subjects is given in Table 1. 

**Table 1:** Demographic characteristics of subjects according to age, sex, occupation, designation, area of work, and work experience ANS: Assistant Nursing Superintendent; DNS: Deputy Nursing Superintendent; NS: Nursing Superintendent; CNO: Chief Nursing Officer

S. No	Variables	Frequency	Percentage
1	Age		
	< 30 Years	222	73.8%
	31-40 Years	76	25.2%
	41- 50 years	3	1%
2	Sex		
	Males	206	68.4%
	Females	95	31.6
3	Occupation		
	Doctors	81	26.9%
	Nurses	220	73.1%
4	Designation		
	Professor/ Associate professor/ Assistant Professor	5	1.7%
	Senior Resident/ Junior resident	76	25.2%
	Nursing officers	189	62.8%
	Senior nursing officers	29	9.6%
	ANS/DNS/NS/CNO	2	0.7%
5	Area of work/posting		
	Intensive care unit	72	23.9%
	Outpatient departments	14	4.7%
	Operation Theatre	29	9.6%
	General wards	143	47.5%
	Emergency department	19	6.3%
	Others	24	8%
6	Total experience in years		
	< 1 year	65	21.6%
	1-5 years	160	53.2%
	5-10 years	63	20.9%
	>10 years	13	4.3%
7	Experience in the present institute in years		
	< 1 year	137	45.6%
	1-5 years	159	52.8%
	5-10 years	4	1.3%
	>10 years	1	0.3%

A large proportion of the study participants (n=271, 90%) received training related to PPE use and 268 (89%) reported that their organization had a policy regarding PPE use. The majority of the study participants (n=257, 85.4%) reported that they are self-motivated and were using appropriate PPE. Most of the participants (n=204, 67.8%) hadn’t acquired any type of infection from the workplace. Description of study subjects according to the type of training regarding PPE use, factors considered while using PPE, and type of PPE frequently used is shown in Table 2

**Table 2:** Type of training regarding PPE use, factors considered while using PPE, and type of PPE regularly used during patient care PPE: personal protective equipment

S. No	Variables		Frequency	Percentage
1.	Training related to PPE use			
	Yes		271	90%
	No		30	10%
2.	Date of training			
	Less than 3 months ago		39	14.4%
	3-6 months ago		35	12.9%
	More than 6 months		197	72.7%
3.	Type of training			
	Training on social media platforms like WhatsApp, Facebook		56	20.7%
	In-person seminars/ workshops		87	32.1%
	In-person on-the-job training		88	32.4%
	Online training		40	14.8%
4.	Factors considered while using PPE			
	Work type	Yes	280	93%
		No	21	7%
	Patient type	Yes	278	92.4%
		No	23	7.6%
	Hazard type	Yes	273	90.7%
		No	28	9.3%
	Institutional policy	Yes	269	89.4%
		No	32	10.6%
5.	Type of PPE regularly used during patient care			
	Surgical mask	Yes	275	91.4%
		No	26	8.6%
	N-95 mask	Yes	289	96%
		No	12	4%
	Gown/Coverall	Yes	221	73.4%
		No	80	26.6%
	Goggles/Face shield	Yes	202	67.1%
		No	99	32.9%
	Shoe cover	Yes	250	83.1%
		No	51	16.9%

In the present study median score for compliance with PPE use among HCWs was found to be 22 with an IQR of 16-24. The median attitude score towards PPE was found to be 42 with an IQR of 16-24. Most of the HCWs (n=226, 75.1%) had favourable attitudes, 56 (18.6%) had unfavourable attitudes, and 19 (6.3%) had neutral attitudes towards PPE use. The current study also assessed various facilitating factors and barriers related to PPE use among HCWs. Table 3 and Table 4 show the frequency and percentage distribution of facilitating factors and barriers related to the appropriate use of PPE among HCWs. 

**Table 3:** Frequency and percentage distribution of various facilitating factors related to PPE use PPE: personal protective equipment

S.N	Item	Frequency (Percentage)
1	Easy handling/ wearing	207 (68.8%)
2	Institutional policy/ guidelines/ protocols	285 (94.7%)
3	Disciplinary action is taken for not following institutional policy for PPE use.	223(74.1%)
4	High Standard or quality of PPE	240 (79.7%)
5	Nature of the exposure anticipated/ obvious in the working environment	282 (93.7%)
6	Adequate information and guidelines are provided on PPE use	273 (90.7%)
7	Availability of PPE	248 (82.4%)

**Table 4:** Frequency and percentage distribution of various barriers related to PPE use PPE: personal protective equipment

S.N	Item	Frequency (Percentage)
1	Inappropriate size	224 (74.4%)
2	Lack of adequate training	166 (55.1%)
3	Discomfort while using PPE	225 (75.8%)
4	Workload and busy schedule	217 (72.1%)
5	As trained to perform some procedures without PPE	140 (46.5%)
6	Handling emergencies	230 (76.4%)
7	The culture of the organization allows non-adherence to PPE protocols	150 (49.8%)
8	If the risk of infection is low	178 (59.1%)
9	Forgetfulness	116 (38.5%)
10	Using PPE makes work harder	219 (72.8%)
11	If co-workers are not complying with PPE protocols	139 (46.2%)
12	Guidelines for the use of PPE are vague	147 (48.8%)
13	Long duty hours	214 (71.1%)
14	Health problems while using PPE	223 (74.1%)
15	Do not have fear of infections	129 (42.9%)
16	Not aware about importance	111 (36.9%)

Univariate quantile regression analysis (simple quantile regression) and multiple quantile regression analysis were done to identify factors influencing compliance with PPE use and details are shown in Table 5 and Table 6. The multiple quantile regression (Table 6) revealed that the variables such as occupation (p<0.001), institutional policy/guidelines/protocols (p=0.003), quality of PPE (p=0.002), availability of PPE (p<0.001), and improper size (p=0.042) were significantly associated with compliance to PPE by HCWs. As compared to nurses, doctors had two points less average score of compliance, which was found to be significant. Availability of PPE, institutional policy, and quality of PPE were significantly associated with higher compliance with PPE use. However, inappropriate size was associated with significantly reduced compliance scores (Table 6).

**Table 5:** Univariate regression analysis (simple quantile regression) with facilitating factors and barriers of PPE use and baseline variables Q1: quartile 1; Q3: quartile 3; PPE: personal protective equipment

Variables		Compliance score			Univariate analysis: Regression coefficient (p-value)
		Median	Q_1_	Q_3_	
Occupation	Doctors	19.00	15.00	23.00	-3 [p<0.001*]
	Nurses	22.00	20.00	24.00	-
Sex	Male	22.00	16.00	24.00	1 [ p=0.183]
	Female	21.00	16.00	23.00	-
Training	Yes	22.00	16.00	24.00	2 [p=0.086]
	No	20.50	16.00	23.00	-
Availability of policy on PPE use in the working institute	Yes	22.00	16.00	24.00	0 [p= 1.000]
	No	22.00	16.00	24.00	-
Previous history of work-related infections	Yes	22.00	16.00	24.00	0 [ p=1.000]
	No	22.00	16.00	24.00	-
Experienced any health problems related to PPE use	Yes	21.50	16.00	24.00	-1 [p=0.156]
	No	22.00	18.00	24.00	-
Easy handling /Wearing	Yes	22.00	16.00	24.00	1 [p=0.184]
	No	21.00	16.00	23.00	-
Institutional policy/ guidelines/ protocols	Yes	22.00	16.00	24.00	4 [p=0.010*]
	No	19.00	13.00	24.00	-
If disciplinary action is taken for not following institutional policy for PPE use.	Yes	22.00	18.00	24.00	1[p=0.209]
	No	21.00	16.00	23.00	-
High Standard or quality of PPE	Yes	22.00	19.00	24.00	4 [p<0.001*]
	No	18.00	15.00	22.00	-
Nature of the exposure anticipated/ obvious in the working environment	Yes	22.00	16.00	24.00	3 [p=0.037*]
	No	19.00	15.00	24.00	-
If adequate information and guidelines are provided on PPE use	Yes	22.00	16.00	24.00	3 [p=0.013*]
	No	19.00	14.50	22.00	-
Availability of PPE	Yes	22.00	19.00	24.00	5[p=<0.001*]
	No	17.00	16.00	21.00	-
Inappropriate size	Yes	21.00	16.00	24.00	-2 [p=0.002*]
	No	23.00	21.00	24.00	-
Lack of adequate training	Yes	22.00	16.00	24.00	0 [p=1.000]
	No	22.00	19.00	24.00	-
Discomfort while using PPE	Yes	22.00	16.00	24.00	-1 [p=0.213]
	No	22.50	19.50	24.00	-
Workload and busy schedule	Yes	22.00	16.00	24.00	0 [p=1.000]
	No	22.00	19.50	24.00	-
As trained to perform some procedures without PPE	Yes	22.00	16.00	24.00	0 [p=1.000]
	No	22.00	19.00	24.00	-
Handling emergencies	Yes	22.00	16.00	24.00	0 [p=1.000]
	No	22.00	16.00	24.00	-
Culture of the organization allows non adherence with PPE protocols	Yes	22.00	16.00	24.00	0 [p=1.000]
	No	22.00	18.00	23.00	-
If risk of infection is low	Yes	22.00	16.00	24.00	1 [p=0.159]
	No	21.00	16.00	23.00	-
Forgetfulness	Yes	23.00	16.00	24.00	1 [p=0.163]
	No	22.00	18.00	23.00	-
Using PPE makes work harder	Yes	22.00	16.00	24.00	0 [p=1.000]
	No	22.00	19.00	24.00	-
If co-workers are not complying with PPE protocols	Yes	22.00	16.00	24.00	0 [p=1.000]
	No	22.00	16.00	23.00	-
Guidelines for the use of PPE are vague	Yes	23.00	16.00	24.00	1 [p=0.152]
	No	22.00	18.00	23.00	-
Long duty hours	Yes	22.00	16.00	24.00	-1 [p=0.194]
	No	23.00	20.00	24.00	-
Health problems while using PPE	Yes	22.00	16.00	24.00	0 [p=1.000]
	No	22.00	18.00	24.00	-
Do not have fear of infections	Yes	23.00	16.00	24.00	1 [p=0.156]
	No	22.00	16.00	23.00	-
Not aware about importance	Yes	23.00	16.00	24.00	1 [p=0.167]
	No	22.00	18.00	24.00	-

**Table 6:** Multiple quantile regression analysis to assess factors influencing compliance of healthcare workers with PPE use Q1: quartile 1; Q3: quartile 3; PPE: personal protective equipment

Variables		Compliance score			Multiple Regression coefficient (p-value)
		Median	Q_1_	Q_3_	
Occupation	Doctors	19.00	15.00	23.00	-2 [p<0.001*]
	Nurses	22.00	20.00	24.00	
Institutional policy/ guidelines/ protocols	Yes	22.00	16.00	24.00	3 [p=0.003*]
	No	19.00	13.00	24.00	-
High standard or quality of PPE	Yes	22.00	19.00	24.00	2 [p=0.002*]
	No	18.00	15.00	22.00	-
Nature of the exposure anticipated/ obvious in the working environment	Yes	22.00	16.00	24.00	1 [p=0.256]
	No	19.00	15.00	24.00	-
If adequate information and guidelines are provided on PPE use	Yes	22.00	16.00	24.00	1 [p=0.248]
	No	19.00	14.50	22.00	-
Availability of PPE	Yes	22.00	19.00	24.00	3 [p<0.001*]
	No	17.00	16.00	21.00	-
Inappropriate size	Yes	21.00	16.00	24.00	-1 [p=0.042*]
	No	23.00	21.00	24.00	-

## Discussion

Standard and transmission-based precautions are an integral part of infection control protocols in a healthcare organization and topmost priority must be given to strict adherence to these infection prevention and control protocols by HCWs caring for patients with infectious diseases to prevent the transmission of infection and to ensure the safety of HCWs. A varied range of factors determines the appropriate and accurate use of PPE. Inappropriate use of PPE and other problems with PPE increases the risk of healthcare-associated infections among HCWs by self-contamination. The current study carried out among 301 HCWs identified numerous factors affecting adherence to PPE use and identified factors contributing to gaps in practice related to PPE use. The present study revealed a high median PPE compliance score of 22 with an IQR of 16-24. High compliance level of PPE has been identified among HCWs during the outbreaks of SARS in 2003 [12] and H1N1 influenza in 2009. [13,14] However, incongruent findings of reduced adherence levels to PPE use were also reported in previous studies [15].

Concerning the level of compliance, nurses reported a better compliance level than other HCWs. The better performance level and skill for the utilization of PPE have been identified in the previous literature [2,10.16]. The reason for this incongruity of findings is not clear, yet a greater participation of nurses in the study as compared to medical doctors might have influenced the findings. The current study identified the availability of PPE and institutional policy on PPE as significant factors influencing compliance with PPE among HCWs. Concurrent findings were reported in previous studies [13,16]. A study carried out on Chinese critical care clinicians during the 2009 H1N1 influenza pandemic identified factors such as the availability of PPE and perceived reprimand for noncompliance as independent predictors of high compliance [13]. A study carried out in paediatric emergency departments in Canada identified PPE being available and convenient as the strongest factor influencing the decision to wear PPE [17]. Poor access and unavailability of PPE are the main factors facilitating the inaccurate use of PPE [18,19]. Organizational factors like the availability of PPE, high work pressure and workload, and limiting time for patient care were associated with the use of PPE [20]. Reduced incidence of mental health problems like depression and anxiety have been documented among nurses having access to appropriate and adequate PPE [21].

In addition to this, improper size of the PPE has been identified as a significant factor affecting inconsistent use of PPE among HCWs. Improper size may hinder the performance of various procedures and increases occupational exposure of HCWs to hazardous agents. Concurrent findings have been reported in a study carried out to identify barriers to using PPE during the COVID-19 outbreak in China [22]. Similarly, a study carried out in Korea during the outbreak of MERS also reported inappropriate and ill-fitting PPE as a major difficulty encountered by HCWs [23]. Quality and standard of PPE are also significant factors influencing compliance. Fan et al. also reported doubts regarding the effectiveness and quality of PPE as a major concern for HCWs during the COVID-19 pandemic [22]. Poor comfort with PPE was a major factor determining the compliance level among HCWs. Overheating with coveralls and fogging and limited visibility due to the use of goggles and N-95 masks were reported as the reasons for reduced compliance [12]. A study carried out among nurses working in intensive care units during the COVID-19 pandemic identified discomfort with PPE due to inappropriate size as a major factor that hinders the performance of nursing procedures and limits the activity of nurses [24]. In the current study, many of the participants (72.8%) felt that using PPE interferes with patient care and causes inconvenience. The inconvenience has been reported as a major factor influencing adherence to PPE in previous studies too [14,25].

Furthermore, accurate and correct access to information about PPE use has also been considered a critical factor influencing adherence to PPE. In the present majority of the participants (90%) reported access to information on the use of PPE and the availability of guidelines as a facilitating factor for ensuring compliance with PPE. These findings were consistent with the findings of cross-sectional studies carried out in Pakistan and Italy during COVID-19 [18,26]. A systematic review also suggested unclear guidelines and improper communication as hindering factors in ensuring HCWs' adherence to IPC protocols [27]. Another study done during the outbreak of MERS reported concordant findings and confusion from unstandardized protocols was identified as a major difficulty for the appropriate use of PPE [23]. Many studies have shown a link between HCWs' risk perception and the adoption of protective health behaviours. But in the current study, HCWs' risk perception was not identified as a predicting factor for ensuring their adherence to PPE use. However, HCWs who were perceived as having a higher risk of getting COVID-19 adopted positive behaviours in a study carried out in Pakistan [18]. Likewise, the decision to use PPE was related to risk perception in a qualitative study conducted to understand factors influencing the utilization of PPE [28]. Risk perception related to contracting the infection from the work environment was influenced by receiving adequate information on PPE use in a study carried out among physcians [26]. Even though knowledge and attitude are considered important factors influencing the behaviour of a person, attitude towards PPE use was not significantly associated with compliance in the present study. These findings were in concordance with Neuwirth et al. [11] and Mokhtari et al. [20]. Neuwirth et al. reported a low level of adherence to PPE despite the awareness regarding the importance of PPE among HCWs in non-COVID-19 wards [11]. Personal factors such as knowledge, attitude, and beliefs were not associated with the use of PPE in a study carried out among nurses during COVID-19 [20]. However, contrasting findings of an association between attitude and knowledge and a strong level of compliance with PPE were reported in an earlier study [13].

Healthcare organizations must consider strategies for continuous training of HCWs to use PPE as a part of pandemic response programmes [29]. Moreover, a survey carried out in India during the early phase of the COVID-19 pandemic to assess the state of PPE preparedness suggested the initiation of quality improvement measures focussing on regular training of HCWs on PPE use improve pandemic preparedness and safe practices [30]. Additionally, supporting HCWs should be a top public health priority and strategies must be implemented to protect them during the pandemic period. [19] 

Although the current study assessed various factors influencing HCWs' compliance with PPE use, this study has some limitations. Self-report bias cannot be excluded as the measurement of all variables in the study was based on self-report measures. So, further studies can be carried out using more objective measures and direct observation to get more realistic information on actual behaviour and adherence to PPE use by HCWs. The convenient sampling technique used for the study can limit the generalizability of findings. Future studies can be carried out to explore the difficulties and barriers encountered with the use of PPE in detail to generate robust evidence to develop gold standard protocols and policies to ensure HCW and patient safety and protection to fight future pandemic outbreaks. Barriers to consistent and effective use of PPE by HCWs can be minimized by understanding, articulating, and implementing various measures at the individual and organizational levels.

## Conclusions

The current study carried out to assess factors influencing compliance with PPE among HCWs identified the availability of institutional policy and guidelines on PPE use, standard or quality of PPE, availability of PPE, and inappropriate size as significant predictors of PPE compliance. Findings put light into initiating various measures and guidelines by government and regulatory bodies to ensure the availability of high-quality PPE along with constructive and consistent feedback and training to adhere to infection prevention and control policies and protocols by HCWs to safeguard themselves and patients from healthcare-associated infections. 

## References

[1] World Health Organization (2022). Rational Use of Personal Protective Equipment For Coronavirus Disease (Covid-19) And Considerations During Severe Shortages. https://www.who.int/publications/i/item/rational-use-of-personal-protective-equipment-for-coronavirus-disease-(covid-19)-and-considerations-during-severe-shortages.

[2] Alao MA, Durodola AO, Ibrahim OR (2020.). Assessment of health workers’ knowledge, beliefs, attitudes, and use of personal protective equipment for prevention of COVID-19 infection in low-resource settings. Adv Public Heal.

[3] George TJ, Shafqat N, Verma R, Bali S (2021). Handling the pandemic our way: a qualitative content analysis of the guidelines issued by Apex Institutes of National Importance (INIs) of India to combat COVID-19 crisis. J Family Med Prim Care.

[4] Nguyen LH, Drew DA, Graham MS (2020). Risk of COVID-19 among front-line health-care workers and the general community: a prospective cohort study. Lancet Public Health.

[5] Brooks SK, Greenberg N, Wessely S, Rubin GJ (2021). Factors affecting healthcare workers' compliance with social and behavioural infection control measures during emerging infectious disease outbreaks: rapid evidence review. BMJ Open.

[6] Honda H, Iwata K (2016). Personal protective equipment and improving compliance among healthcare workers in high-risk settings. Curr Opin Infect Dis.

[7] Atkinson P, French J, Lang E, McColl T, Mazurik L (2020). Just the Facts: protecting frontline clinicians during the COVID-19 pandemic. CJEM.

[8] Wang J, Zhou M, Liu F (2020). Reasons for healthcare workers becoming infected with novel coronavirus disease 2019 (COVID-19) in China. J Hosp Infect.

[9] Cook TM (2020). Personal protective equipment during the coronavirus disease (COVID) 2019 pandemic - a narrative review. Anaesthesia.

[10] Okello TR, Kansime K, Odora J (2017). Barriers and factors affecting personal protective equipment usage in St. Mary’s Hospital Lacor in Northern Uganda. East Cent African J Surg.

[11] Neuwirth MM, Mattner F, Otchwemah R (2020). Adherence to personal protective equipment use among healthcare workers caring for confirmed COVID-19 and alleged non-COVID-19 patients. Antimicrob Resist Infect Control.

[12] Wilder-Smith A, Chiew CJ, Lee VJ (2020). Can we contain the COVID-19 outbreak with the same measures as for SARS?. Lancet Infect Dis.

[13] Hu X, Zhang Z, Li N (2012). Self-reported use of personal protective equipment among Chinese critical care clinicians during 2009 H1N1 influenza pandemic. PLoS One.

[14] Daugherty EL, Perl TM, Needham DM, Rubinson L, Bilderback A, Rand CS (2009). The use of personal protective equipment for control of influenza among critical care clinicians: a survey study. Crit Care Med.

[15] McGaw CD, Tennant I, Harding HE, Cawich S, Crandon I, Walters C (2012). View of healthcare workers’ attitudes to and compliance with infection control guidelines in the operating department at the university hospital of the West Indies, Jamaica. Int J Infect Control.

[16] Moore D, Gamage B, Bryce E, Copes R, Yassi A (2005). Protecting health care workers from SARS and other respiratory pathogens: organizational and individual factors that affect adherence to infection control guidelines. Am J Infect Control.

[17] Reid SM, Farion KJ, Suh KN, Audcent T, Barrowman NJ, Plint AC (2011). Use of personal protective equipment in Canadian pediatric emergency departments. CJEM.

[18] Hakim M, Khattak FA, Muhammad S (2021). Access and use experience of personal protective equipment among frontline healthcare workers in Pakistan during the COVID-19 emergency: a cross-sectional study. Health Secur.

[19] Delgado D, Wyss Quintana F, Perez G, Sosa Liprandi A, Ponte-Negretti C, Mendoza I, Baranchuk A (2020). Personal safety during the COVID-19 pandemic: realities and perspectives of healthcare workers in Latin America. Int J Environ Res Public Health.

[20] Mokhtari R, Safdari A, Hekmatpou D, Sahebi A, Moayedi S, Torres M, Golitaleb M (2021). Investigating the effective factors of using personal protective equipment from the perspective of nurses caring for COVID-19 patients: a cross-sectional study. Int J Environ Res Public Health.

[21] Arnetz JE, Goetz CM, Sudan S, Arble E, Janisse J, Arnetz BB (2020). Personal protective equipment and mental health symptoms among nurses during the COVID-19 pandemic. J Occup Environ Med.

[22] Fan J, Jiang Y, Hu K (2020). Barriers to using personal protective equipment by healthcare staff during the COVID-19 outbreak in China. Medicine (Baltimore).

[23] Kang J, Kim EJ, Choi JH (2018). Difficulties in using personal protective equipment: training experiences with the 2015 outbreak of Middle East respiratory syndrome in Korea. Am J Infect Control.

[24] Jose S, Cyriac MC, Dhandapani M (2021). Health problems and skin damages caused by personal protective equipment: experience of frontline nurses caring for critical COVID-19 patients in intensive care units. Indian J Crit Care Med.

[25] Seitz RM, Yaffee AQ, Peacock E, Moran TP, Pendley A, Rupp JD (2021). Self-reported use of personal protective equipment among emergency department nurses, physicians and advanced practice providers during the 2020 COVID-19 pandemic. Int J Environ Res Public Health.

[26] Savoia E, Argentini G, Gori D, Neri E, Piltch-Loeb R, Fantini MP (2020). Factors associated with access and use of PPE during COVID-19: a cross-sectional study of Italian physicians. PLoS One.

[27] Verbeek JH, Rajamaki B, Ijaz S (2020). Personal protective equipment for preventing highly infectious diseases due to exposure to contaminated body fluids in healthcare staff. Cochrane Database Syst Rev.

[28] Harrod M, Weston LE, Gregory L, Petersen L, Mayer J, Drews FA, Krein SL (2020). A qualitative study of factors affecting personal protective equipment use among health care personnel. Am J Infect Control.

[29] Piché-Renaud PP, Groves HE, Kitano T (2021). Healthcare worker perception of a global outbreak of novel coronavirus (COVID-19) and personal protective equipment: survey of a pediatric tertiary-care hospital. Infect Control Hosp Epidemiol.

[30] Haji JY, Subramaniam A, Kumar P, Ramanathan K, Rajamani A (2020). State of personal protective equipment practice in Indian intensive care units amidst COVID-19 pandemic: a nationwide survey. Indian J Crit Care Med.

